# Impact of Human Body Temperature on Stress Tolerance and Transcriptome of *Cronobacter sakazakii*

**DOI:** 10.3390/pathogens14030281

**Published:** 2025-03-14

**Authors:** Siqi Li, Yuanyuan Wang, Yahao Yang, Xinlu Yu, Jiajia Liu, Meiling Jiang, Jing Zhang, Ge Yun, Yufei Han, Heng Wang, Qiong Xie, Gukui Chen

**Affiliations:** 1 School of Medicine, Northwest University, Xi’an 710069, China; 202233068@stumail.nwu.edu.cn (S.L.); 202233020@stumail.nwu.edu.cn (Y.Y.); 202332730@stumail.nwu.edu.cn (X.Y.); 202233025@stumail.nwu.edu.cn (J.L.); 202332757@stumail.nwu.edu.cn (M.J.); 202332705@stumail.nwu.edu.cn (J.Z.); 202422217@stumail.nwu.edu.cn (G.Y.); 15628309202@163.com (Y.H.); 2 College of Medical Technology, Shaanxi University of Chinese Medicine, Xianyang 712046, China; wyy2589@163.com; 3 China Astronaut Research and Training Center, Beijing 100086, China; aaasss3627@outlook.com; 4 Key Laboratory of Resources Biology and Biotechnology in Western China, Ministry of Education, Northwest University, Xi’an 710069, China; 5 ShaanXi Provincial Key Laboratory of Biotechnology, Xi’an 710069, China

**Keywords:** foodborne pathogen, growth temperature, acid resistance, osmotic stress tolerance, transcriptional regulation

## Abstract

*Cronobacter sakazakii* is a food-borne pathogen that can thrive in various environments, including the human body. The human body’s physiological temperature exceeds that of the environment (22–30 °C), necessitating adaptations to heat stress during this transition. Managing heat stress is crucial when transitioning from the environment to the human body. In this study, we explored the effect of human body temperature on the growth of planktonic *C. sakazakii*, as well as its acid resistance, osmotic stress resistance, autoaggregation, and cell surface hydrophobicity. Our study demonstrated that human body temperature facilitated the growth, acid resistance, and osmotic resistance of *C. sakazakii*, compared to 28 °C. The relationship between human body temperature and phenotypes was studied by comparing gene expression at human and environmental temperatures (37 to 28 °C) using high-throughput sequencing. The results revealed up-regulation in the expression of 626 genes, including genes involved in arginine and proline metabolism, carbon fixation pathways, and nitrogen metabolism. Further analysis showed that human body temperature is essential for the environmental stress resistance of *C. sakazakii*. It boosts denitrification, betaine transport, and universal stress proteins, supporting membrane integrity and osmoprotectant transport. This study enhances our understanding of the strategies employed by *C. sakazakii* during its adaptation to the human body.

## 1. Introduction

*Cronobacter sakazakii* is a gram-negative facultative anaerobic bacillus recognized as an emerging neonatal pathogen transmitted through contaminated foods [1], with a high mortality rate (approximately 42–44%) [2]. This pathogen induces bacteraemia, necrotizing enterocolitis, meningitis, and sepsis in neonates and infants, while manifesting wound or urinary tract infections in adult populations [3]. This pathogen is able to multiply at temperatures ranging from 6 to 45 °C [4]. It has been isolated from diverse food and environmental substrates including aquatic systems (river water, underground water, and tap water), aquatic products, meat products, viscera from food-producing animals, milled cereals, cereal products, ready-to-eat food products, and other substrate types. However, the most common vehicles of transmission for this organism are powdered infant formula and powdered milk, due to the strong resistance of *Cronobacter spp*. to drying [5].

A variety of virulence factors have been identified to be associated with the pathogenicity of *C. sakazakii*. Genomic analysis of *C. sakazakii* have identified a metabolism pathway (encoded by the *nanAKT* gene cluster) for sialic acid [6,7]. Since sialic acid is an essential component for brain gangliosides, this metabolism pathway may contribute to the predominance of *C. sakazakii* in neonatal and infant infections [8]. The lipoprotein NlpD functions to maintain the intracellular bacterial pH, facilitating the resistance of *C. sakazakii* to acid stress in the stomach and within macrophages [9]. During host cell invasion and translocation into the deeper organs, two outer membrane proteins, OmpA and OmpX, were reported to play critical roles [7]. Zpx, Cpa, and type III hemolysin Hly are essential for *C. sakazakii* to cause host cell deformation, confer serum resistance, and produce hemolytic activity, respectively [10,11]. Additional virulence factors that have been proposed include flagella-associated genes (*flhD*, *motA*, *flgM*, *flgB*, and *flic*), lipoprotein genes (*slyB*, *blc*, *to1c/A*), and other regulatory factors (*sdiA*, *cheY*, *bss*, *fliT*) [12].

Bacteria must sense and respond to changes in environmental cues, such as the availability of oxygen, altered osmolarity, or pH [13,14]. Temperature is one of the important signals that a mammalian pathogen uses to regulated the virulence trait once it has entered its warm-blooded host [15]. Thermal regulation of gene expression has been described for several facultative pathogens. For example, expression and assembly of the type III secretion system (T3SS) encoded on the virulence plasmid of *Yersinia pseudotuberculosis* is induced at 37 °C in an RNA thermometer-dependent manner [16]. A number of *Pseudomonas aeruginosa* virulence factors are regulated by temperature, including T3SS, quorum sensing, and phenazine [17]. Exposure to human body temperature impacts the virulence and cell morphology of the pathogen *Photobacterium damselaer* [18].

However, the regulatory role of human body temperature in the virulence, acid resistance, and osmotic stress tolerance remains to be fully explored in *C. sakazakii*. In this study, to understand the phenotypes, mechanistic basis, and factors involved in the successful adaptation of the *C. sakazakii* to human body temperature, we performed a global transcript analysis to generate a high-resolution map of transcription start sites and identify mRNAs and sRNAs for *C. sakazakii* ATCC29544 at 28 and 37 °C using RNA-seq.

## 2. Materials and Methods

### 2.1. Bacterial Strains, Plasmids, and Growth Conditions

Bacterial strains, plasmids, and primers used in this study are listed in Table 1 and Table 2. *Escherichia coli* strains were incubated in Luria-Bertani (LB) medium and *C. sakazakii* strains were incubated in LB at 37 °C.

### 2.2. Construction of Gene Deletion Mutants

The gene deletion mutants were constructed using the λ red recombinase system as previously described [19,20]. Briefly, plasmid pKD46 was transformed into *C. sakazakii* wild-type strain after cold sucrose treatment [20]. Primer pairs for target gene deletion were used to amplify chloramphenicol (Cm)-resistance gene fragments flanked by FRT (FLP recognition target) sites and homologous arms, which were then electrically transferred to WT/pKD46 competent cells prepared by 0.2–0.5% L-arabinose induction and cold sucrose treatment. A positive colony on the Cm-containing LB plate was streaked onto a fresh Cm plate and incubated at 42 °C to eliminate pKD46 plasmid. All primer sequences are listed in Table 2.

### 2.3. Growth Curves

Overnight culture of *C. Sakazakii* ATCC29544 in LB medium at 37 °C or 28 °C was diluted at a ratio of 1:100 into a conical flask containing 100 mL LB medium for further 14 h culture at 37 °C or 28 °C. The growth rate of the bacteria was detected with a spectrophotometer (YOUKE, Shanghai, China), and the optical density (OD) of the bacteria was detected at 600 nm every one hour. Each sample was repeated three times.

### 2.4. Analysis of Acid Resistance

The pH of the medium was adjusted so that the initial pH was pH 7.3, pH 6.0, pH 5.0, pH 5.5, or pH 3.0. The target strains cultured overnight were diluted at a ratio of 1:100, and then cultured at 37 °C or 28 °C. OD600 was measured every 2 h to plot acid-resistant growth curves, which was repeated three times for each sample.

### 2.5. Analysis of Osmotic Stress Tolerance

The single colonies were inoculated in LB medium and cultured overnight at 37 °C or 28 °C. The suspension of each strain was inoculated in LB with different concentrations of NaCl (0%, 3%, 6%, 8%, and 10%) at a ratio of 1:100, and then the samples were cultured at 220 rpm, at 37 °C or 28 °C, for 12 h. The OD600 of the samples was determined by spectrophotometer at 12 h, and each experiment was repeated three times.

### 2.6. Autoaggregation, Cell Surface Hydrophobicity

The wild-type of *C. sakazakii* ATCC29544 was incubated at 37 °C or 28 °C, 220 rpm for 12 h, then washed, suspended in PBS, and the OD600 concentration of the suspension was measured and recorded as A_0_. To assess autoaggregation, 5 mL of heavy suspension was added to the glass tube for incubation at 28 °C or 37 °C without rotation for 24 h. The concentration of the bacterial solution 1 cm below the liquid level was measured and recorded as A_1_. The AAg percentage was calculated as follows:

AAg% = (A_0_ − A_1_)/A_0_ × 100%

To assess cell surface hydrophobicity (CSH), overnight culture of the bacteria strain was pelleted, washed, and resuspended with PBS buffer. The OD600 concentration of the suspension was measured and recorded as A_2_. 800 µL xylene was mixed with the suspensions (2 mL), and then incubated at 28 °C or 37 °C for 1 h. The aqueous phase was measured as A_3_. The surface hydrophobicity index (H%) was calculated as follows:

H% = (A_2_ − A_3_)/A_2_ × 100%

### 2.7. Sample Preparation for RNA-Seq

Overnight cultured bacteria (WT) were added to LB medium at a ratio of 1:100 and shaken to OD600 = 0.8 at a temperature of 37 °C or 28 °C, followed by centrifugation at 8000 rpm for 3 min and two washes with PBS. Next, the bacteria were collected for RNA-Seq analysis. All samples were prepared in triplicate. RNA-Seq analysis was performed by the Shanghai Majorbio Technology (Shanghai, China).

### 2.8. Mapping of RNA-Seq Libraries and Differential Gene Expression Analysis

Clean data (reads) were compared with the reference genome to obtain the mapped data (reads) for subsequent analysis, and the DEseq2 method was used to standardize the readcount data and determine the differential expression of genes based on their readcount value [21]. Then gene expression analysis and RNA-seq correlation analysis were conducted to obtain the differentially expressed genes (DEGs). Finally, GO and KEGG enrichment analysis was performed to compare the DEGs between 37 °C and 28 °C.

### 2.9. Quantitative Real-Time PCR Verification

Several candidate genes with expression differences were randomly selected for quantitative real-time PCR (RT-PCR) analysis, using the specific primers designed by Beacon Designer 8 (Table 2). The total RNA of WT at 37 °C or 28 °C was extracted by the RNAprep pure Bacteria Kit (TIANGEN, Beijing, China), and then transcribed into cDNA by reverse transcriptase (TRANSGEN, Beijing, China). RT-PCR was performed using SuperReal PreMix Plus (SYBR Green) (TIANGEN, Beijing, China) under the conditions of pre-denaturation at 95 °C for 15 min, denaturation at 95 °C for 10 s, annealing at 56 °C for 20 s, extension at 72 °C for 20 s, and 40 cycles were repeated. The product specificity was analyzed by dissolution curve, and the 16S rRNA gene was used as the internal control. Each sample was tested at least three times. The relative gene expression of WT at 37 °C and 28 °C was calculated by the 2^−ΔΔCt^ method.

### 2.10. Statistical Analysis

Each experiment was repeated at least three times. The data were expressed as mean and standard deviations (*n* = 3). Statistical analyses were performed using Student’s *t*-test with SPSS Statistics 16.0. Statistical significance was defined as *p* < 0.05, and significant differences between groups are indicated by a single asterisk (*) in the figures.

## 3. Results

### 3.1. Effect of Temperature on Growth

**Figure 1:**
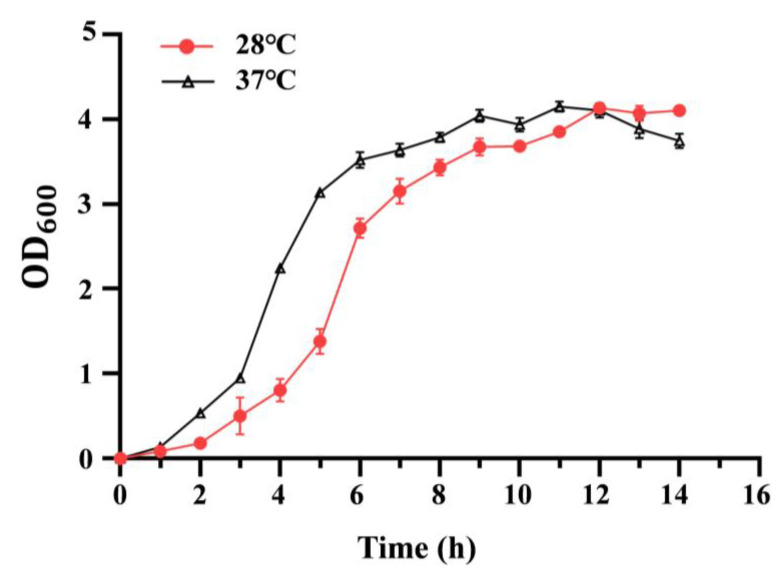
Growth curves of *C. sakazakii* WT strain at 28 °C and 37 °C. Overnight culture was diluted 100-fold into fresh LB medium with aeration. The bacteria’s optical density (OD) was detected at 600 nm every hour. Error bars represent standard deviations, *n* = 3.

To analyze the effect of temperature on bacterial growth, the growth curve of the *C. sakazakii* ATCC29544 strain was determined at 28 °C or 37 °C. As shown in Figure 1, *C. sakazakii* ATCC29544 displayed a faster growth rate at 37 °C than at 28 °C before 12 h. At 12 h, the two growth curves crossed. After 12 h, this strain grew slightly faster at 28 °C than at 37 °C (See Table S1 for details). Additionally, the growth curve of the *C. sakazakii* ATCC29544 strain was determined at 28 °C or 37 °C in both TSB (nutrient-rich medium) and M9 medium (minimal medium), and the growth patterns were consistent with those observed in LB (Figure S1). These results showed that growing temperature significantly affects the growth of the *C. sakazakii* strain. The reversal growth rate before and after 12 h might be due to the faster nutriment consumption and accumulation of toxic substance at 37 °C.

### 3.2. Effect of Temperature on the Acid Resistance

**Figure 2:**
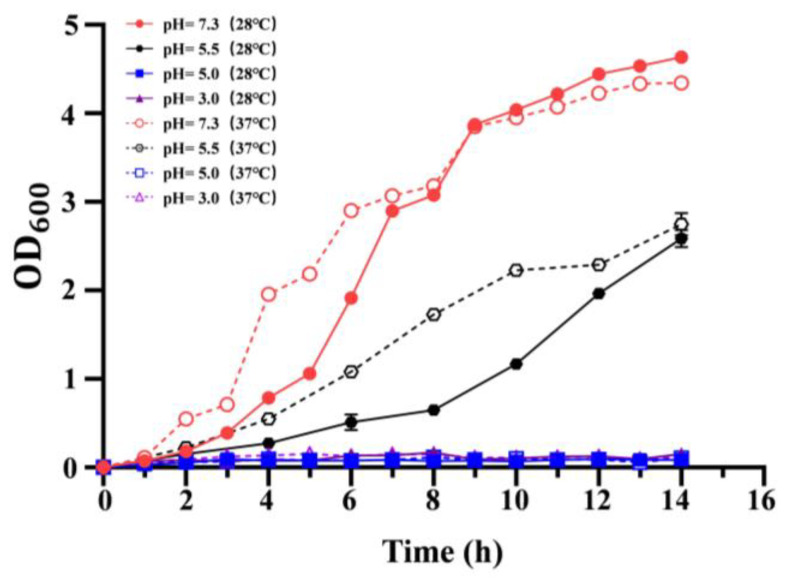
Growth curve of *C. sakazakii* WT strain on LB medium at different pH (pH = 7.3, 5.5, 5.0, 3.0). OD600 was measured every 2 h, which was repeated three times for each sample. Error bars represent standard deviations, *n* = 3.

To evaluate the effect of growth temperature on the acid resistance, the growth of *C. sakazakii* ATCC29544 in LB medium at different pH (7.3, 5.5, 5.0, 3.0) was determined. However, the wild-type strains failed to grow at pH 5.0 and pH 3.0 (Figure 2). Compared to pH 7.3, the growth rate of the wild-type strain decreased significantly at pH 5.5 at 37 and 28 °C. Additionally, at pH 5.5, the growth rate of the wild-type strain at 28 °C was lower than that at 37 °C before 14 h. These results showed that growth temperature significantly influences acid resistance of *C. sakazakii* ATCC29544, and this wild-type strain has a stronger acid resistance at 37 °C.

### 3.3. Effects of Temperature on Growth Under Osmotic Stress

**Figure 3:**
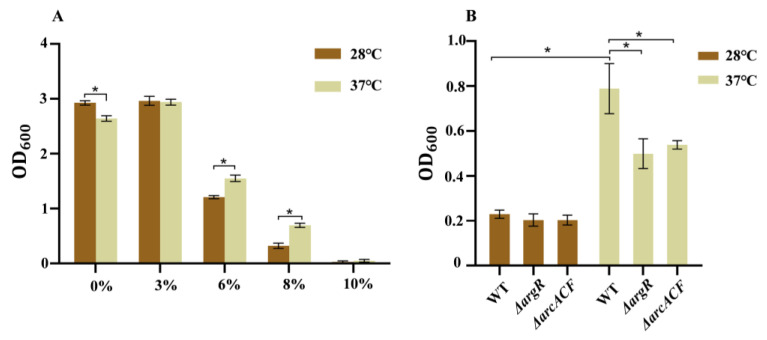
The NaCl resistance of *C. sakazakii* WT at 28 °C and 37 °C. (**A**) WT strains were transferred to LB with different NaCl concentrations (0%, 3%, 6%, 8%, 10%) and cultured for 12 h; (**B**) WT, Δ*argR* and Δ*arcACF* strains were transferred to LB with 8% NaCl concentration and cultured for 12 h. Error bars represent standard deviations, *n* = 3. A representative of three independent experiments with the same results is shown. The data shown are means ± standard errors of the means. * *p* < 0.05 by Student’s *t* test.

Figure 3A represents *C. sakazakii*’s tolerance to NaCl at different temperatures. Overnight culture of the wild-type strain was transferred into LB medium with different NaCl concentrations (0%, 3%, 6%, 8%, 10%). In medium without and with 3% NaCl, the wild-type strain grew fast at both temperatures and grew faster at 28 °C than at 37 °C. When the NaCl concentration was increased (6%, 8%, and 10%), bacterial growth was inhibited at both temperatures. In medium with 6% and 8% NaCl, this strain grew faster at 37 °C than at 28 °C. However, the strain failed to grow in LB medium with 10% NaCl. These results indicate that compared to 28 °C, the wild-type strain displays higher resistance to high osmotic stress (6% and 8% NaCl) at human body temperature.

### 3.4. Effects of Temperature on the Autoaggregation, Cell Surface Hydrophobicity

**Figure 4:**
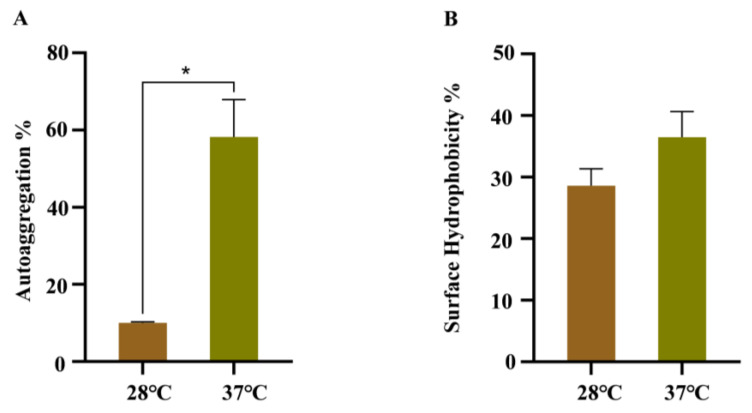
Autoaggregation and cell surface hydrophobicity of WT strains at 28 °C and 37 °C. (**A**) Autoaggregation; (**B**) cell surface hydrophobicity. Error bars represent standard deviations, *n* = 3. A representative of three independent experiments with the same results is shown. The data shown are means ± standard errors of the means. * *p* < 0.05 by Student’s *t* test.

The effects of temperature on the autoaggregation and cell surface hydrophobicity were analyzed, due to their important roles during bacterial cell adhesion. As shown in Figure 4A, WT strain growing at 37 °C has a faster precipitation rate in LB than WT strain growing at 28 °C. The CSH assay suggested that more cells were retained in the water phase of WT strain growing at 28 °C after mixing with xylene, indicating a decrease in CSH (Figure 4B).

### 3.5. RNA-Seq Analysis of WT at 37 °C and 28 °C

**Figure 5:**
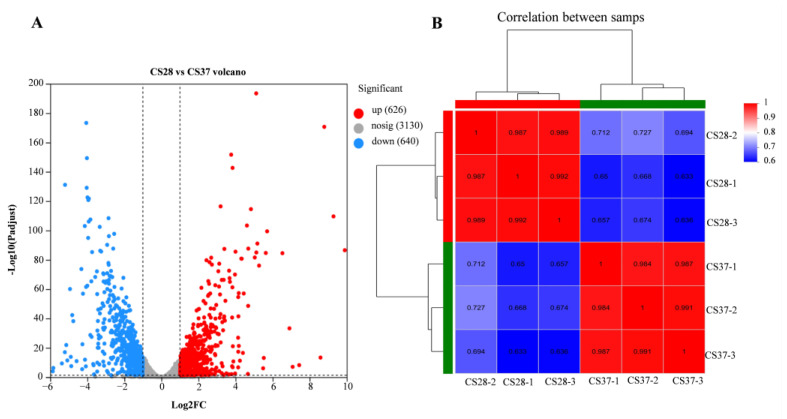
Volcano map of differentially expressed genes and Pearson correlation coefficients between samples. (**A**) Red is a significantly up-regulated gene; blue is a significantly down-regulated gene; gray is for genes that are not significantly expressed. (**B**) The closer the Pearson correlation coefficient between samples is to 1, the higher the similarity. A high value between the biological samples indicated that the samples have good repeatability.

We used RNA-seq to examine the global gene expression of *C. sakazakii* ATCC29544 at 37 °C and 28 °C, with an objective to assess the genes that contribute to adaptation to human body temperature (Table S2). The correlation between biological replicates was evaluated by the Pearson correlation coefficient, which was higher than 0.98 under the same experimental conditions, indicating that the correlation between samples was strong and they could be used for the subsequent experiments (Figure 5). According to the transcription data, the genes with a fold change ≥ 2 and *p* < 0.05 were considered the genes with significant differences in expression. We found that there were 1266 differentially expressed genes (DEGs) (Figure 5), with 626 up-regulated and 640 down-regulated genes at 37 °C compared with 28 °C (Table S3).

**Figure 6:**
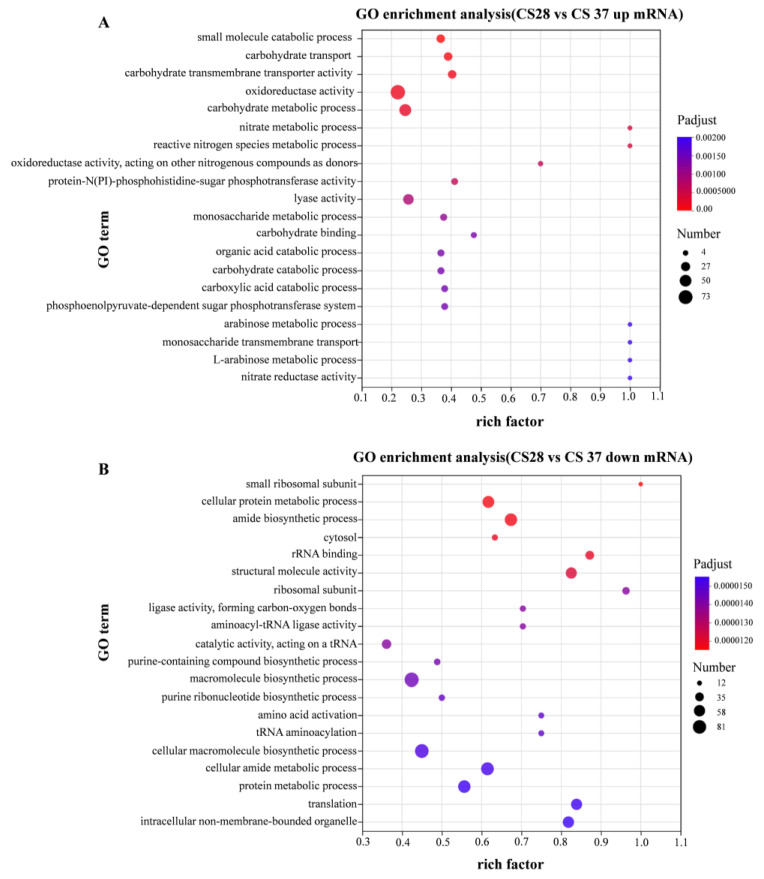
GO enrichment analysis of differentially expressed genes. Compared with WT strain growing at 28 °C, the expression level of strain growing at 37 °C was significantly up-regulated (**A**) and significantly down-regulated (**B**).

Gene ontology (GO) enrichment analysis divided the genes into three functional categories: biological process, cellular component, and molecular function. Figure 6A shows the enrichment GO terms with up-regulation. Gene ontology (GO) enrichment analysis showed significant enrichment for genes related to oxidoreductase activity, carbohydrate metabolic process, and catabolic process among the genes up-regulated at 37 °C. Figure 6B shows the enrichment GO terms with down-regulation, compared with 28 °C; the down-regulated DEGs in 37 °C are significantly enriched in macromolecule biosynthetic process, cellular macromolecule biosynthetic process, and cellular amide metabolic process. To sum up, the DEGs between 28 and 37 °C primarily involve the cellular component.

**Figure 7:**
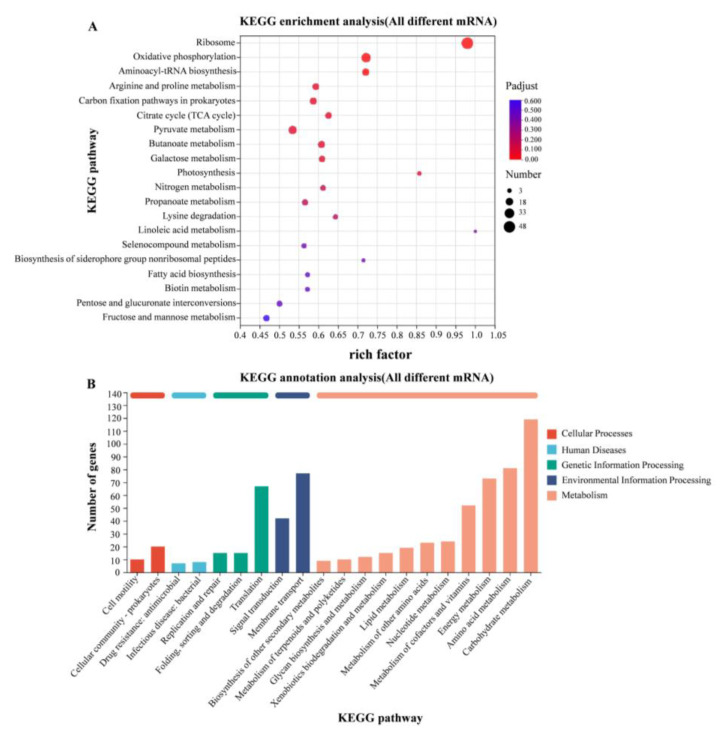
KEGG enrichment analysis of differentially expressed genes. (**A**) KEGG enrichment analysis of differentially expressed genes in WT strains at 28 °C and 37 °C; (**B**) KEGG functional annotation analysis of differentially expressed genes in WT strains at 28 °C and 37 °C.

According to the KEGG gene database, the enriched KEGG metabolic pathways corresponding to the differently expressed proteins were analyzed. Transcriptome analysis revealed significant transcriptional changes in metabolism and environmental information processing. The expression levels of genes involved in amino acid metabolism were up-regulated in 37 °C relative to 28 °C, mainly including arginine deiminase (*arcA*) and carbamate kinase (*arcC*) genes in arginine metabolism (Figure 7).

### 3.6. Verification of RNA-Seq Results by qRT-PCR

**Figure 8:**
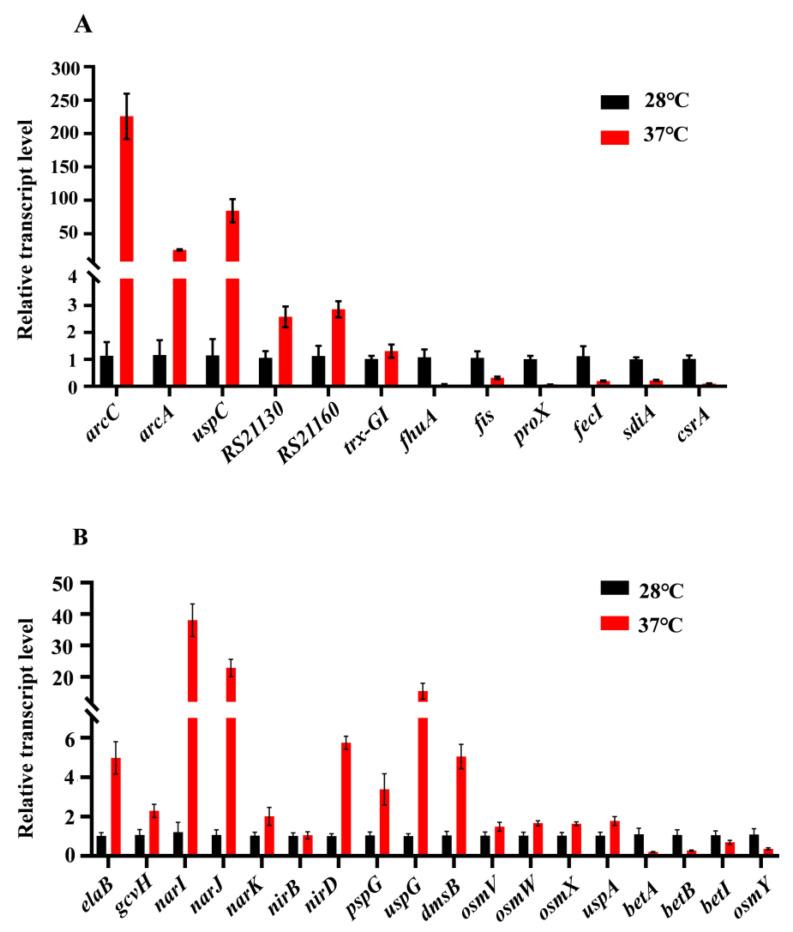
Real-time qRT-PCR. (**A**) The relative expression levels (37 °C compared to 28 °C) of 12 genes related to metabolic and environmental stress tolerance were analyzed, including six down-regulated genes and six up-regulated genes; (**B**) The transcriptional levels (37 °C compared to 28 °C) of genes that may play a role in acid resistance and osmotic stress.

To further validate the results from transcriptomic analysis, qRT-PCR was used to analyze the relative expression levels of 12 genes related to metabolism and environmental stress tolerance, at 37 °C compared to 28 °C, including six down-regulated (*fhuA*, *fis*, *proX*, *fecI*, *sdiA*, and *csrA*) and six up-regulated (*arcC*, *arcA*, *uspC*, *RS21130*, *RS221160*, and *trx-GI*) genes (Figure 8A). Furthermore, we also detected the relative expression levels of some genes that might play roles in acid resistance and osmotic stress (Figure 8B).

### 3.7. Definition of Transcriptional Units and the Analysis of 5′ Untranslated Regions (5′ UTRs)

Revealing the operon organization in bacteria is essential for understanding bacterial RNA-based regulation [22]. We were able to map transcription start sites (TSSs) for 2683 transcriptional units (TU) (Table S4). Of these, 869 were found to be polycistronic TUs. Over 77% of the multi-gene operons are shorter than 4000 bp, and only 2.5% are longer than 8000 bp (Figure S2). This general operon organization is highly similar to that found in other bacteria.

Additionally, since our TSS mapping defines the 5′ ends of TUs, it also reveals the 5′ UTRs of the downstream genes. Analysis of the 5′ UTRs for the 2683 TUs showed a median 5′UTR length less than 50 nt. Most of the 5′ UTRs (63.3%) were no more than 50 nt. However, 163 5′ UTRs were longer than 200 nt (Figure S3).

### 3.8. Detection of Expressed Small RNAs

By combining our TSS mapping and the whole-transcriptome data, we determined 177 events of intergenic transcription by alignment with the current known sRNAs from 4 database (sRNATarBase, sRNAMap, Rfam, and SIPHT) (Table S5). Of these, eight are identified in more than one database and four are homologues of previously well-characterized sRNAs (GlmZ, GcvB, CsrB, and CsrC) from other bacteria. In comparison with the 28 °C group, 64 sRNA candidates were identified as differently expressed in the 37 °C group, of which 24 were up-regulated and 40 were down-regulated (Table S5).

## 4. Discussion

Variation in temperature is one of the most crucial stress factors for pathogens of environmental origin during adaptation to the human body. Therefore, we conducted one of the first studies investigating the response of *C. sakazakii* to the human body temperature (37 °C), in an effort to better understand the factors promoting *C. sakazakii* tolerance and pathogenesis. The capacity of *C. sakazakii* to sense, respond to, and adapt to variable and hostile environmental conditions, such as osmotic stress, acid, heat, oxidation, drying, and bile salts, makes it successful in its natural environment and increases its ability to survive in its host [23,24,25]. In this study, the acid and osmotic stress resistance of *C. sakazakii* were investigated to determine the effect of human body temperature on its environmental tolerance.

Modulation of gene expression by the temperature experienced by microorganisms has been described for a number of facultative pathogens [17,26,27]. Here, we found that several genes involved in arginine metabolism (Figure 7), TCA cycle, and carbon fixation were up-regulated at human body temperature, which is consistent with the faster growth rate at 37 °C compared to 28 °C. Universal stress proteins play a crucial role in helping bacteria resist various environmental pressures, including high temperature, intracellular immune responses, oxidative stress, low pH, osmotic stress, and antibiotics [28]. In *E. coli*, the UspA gene was up-regulated under high temperature conditions to resist temperature stress [29]. Usp1 gene expression is up-regulated with increasing temperature in *Burkholderia cepacia* [30]. UspA in *Salmonella* is induced and up-regulated under metabolic, oxidative, and temperature stresses [31]. In this study, transcriptomic data showed that several Usp genes, such as *uspA*, *uspC*, and *uspG* were also up-regulated at human body temperature.

The locus of heat resistance (LHR) is a genomic island flanked by mobile genetic elements, which transfers among diverse species of enterobacteriaceae, including opportunistic human pathogens in the genera *Cronobacter*, *Klebsiella*, and *Enterobacter* [32,33]. LHR confers resistance to extreme heat, osmotic stress, chlorine, and oxidative stress in *E. coli* and in *C. sakazakii* [34,35]. It was reported that expression of LHR genes was induced at high temperature (54 °C) without shaking in *C.sakazakii* [34]. In the present study, growth at human body temperature led to induction in the expression of LHR genes. The qRT-PCR analysis showed that the relative expression of CSK29544_*RS21130* and CSK29544_*RS21160* was induced to 5.2- and 4.45-folds at human body temperature, respectively.

Foodborne pathogens have evolved a variety of acid-tolerance genes for survival in acidic environments [9,36,37,38,39]. Although many studies have identified a series of acid determinants for *C. sakazakii*, the effect of human body temperature on acid-resistance remains elusive. In our work, we found that the *C. sakazakii* ATCC29544 strain failed to survive at pH levels of 3.0 and 5.0. However, at pH 5.5, the growth rate was faster at 37 °C than that at 28 °C. The low pH of gastric secretions is considered the body’s first line of defense against food-borne pathogens. Since the gastric pH of infants varies from 2.9 to 5.8 [40], our results indicate that growth at human body temperature protects *C. sakazakii* from being killed during its passage through the stomach [41].

When encountering an acidic environment, the cytoplasmic membrane integrity and proton motive force are destroyed, resulting in excessive intracellular protons and inhibited growth of bacteria. We found that the expression of the *elaB* gene was increased at 37 °C, which indicated that ElaB may facilitate bacterial acid tolerance by promoting membrane integrity at human body temperature [42]. In our results, expression of the *pspG* gene, one component of the phase shock response system (Psp), was also increased significantly at 37 °C. The Psp is another important system that may be involved in virulence and acid resistance in *C. sakazakii*, since it has been reported to be essential for virulence in *Yersinia enterocolitica* and induced by a variety of environmental cues that could have a negative impact on the inner membrane (IM), such as extreme temperatures, osmolarity, and ethanol [43,44]. Additionally, proton consumption through metabolic reactions is another strategy for bacteria to maintain in acidic environments.

The expression of genes *arcC, argF*, and *arcA* involved in the arginine metabolism pathway were up-regulated drastically by 9.89, 9.29, and 8.79 lgo_2_ FC at 37 °C, respectively (Table S4). Previous reports suggested that *C. sakazakii* consumes protons mainly through the arginine deiminase system under acid conditions [36]. Arginine metabolism via the arginine deiminase pathway is conserved in various bacteria and provides protection for the survival and growth of bacteria in acidic environments [45,46]. This pathway converts arginine into ammonia, CO_2_, and ornithine, accompanied by ATP production, which consumes protons by binding to ammonia to form ammonium ions, and then increases the pH to enhance its acid stress resistance. The reason why the arginine deiminase system is the preferred acid-resistance system may be that it not only consumes protons but also produces energy [47]. We constructed the Δ*argR* and Δ*arcACF* mutants (Figure S4) and revealed that inactivation of the arginine deiminase system had no obvious effect on acid resistance (Figure S5). This indicates that multiple factors collectively contribute to the increased acid resistance at human body temperature. For example, genes involved in denitrification, such as *narI*, *narJ*, *narK*, *nirB*, and *nirD* were all up-regulated at 37 °C.

Genes encoding another system, the glycine cleavage system GcvPTH, were also up-regulated to varying degrees, in which *gcvH* was up-regulated by 3.3-folds. This system catalyzes the decarboxylation of glycine to consume protons and produce carbon dioxide, ammonia, and methylene tetrahydrofolate, which is also important for the adaptability of bacteria under adverse environments [48,49]. In addition, several other genes that affect acid resistance were also up-regulated at human body temperature, such as *uspA* (universal stress protein A) [50,51] and *dmsB* (dimethylsulfoxide reductase) [36].

Osmotic stress is typically encountered by *C. sakazakii* during food production (especially in powdered infant formula) [52]. The ability of *C. sakzakii* to withstand osmotic stress is important to its survival. At human body temperature, *C.sakazakii* grew faster in mediums with 6% and 8% NaCl (Figure 3). Betaine has been applied as one of the osmoprotectants due to its special methylation-related structure. However, transcriptome data revealed that human body temperature has no significant effect on the expression of betaine biosynthesis genes *betI*, *betB*, and *betA*. Glycine betaine is more effective than other osmolytes for increasing osmolality in water or concentrated protein solutions in vitro. The major betaine transport proteins identified in bacteria are ProP, ProVWX (ProU system), and OsmVWXY (OsmU system) [53]. Expression of *proP* showed no change at 37 °C, whereas expression of *proVWX* genes was significantly decreased. On the contrary, expression of the third betaine transporter OsmU was slightly increased at human body temperature, consistent with our phenotypic result. In addition to betaine biosynthesis and transporter genes, other genes previously shown to be involved in osmotic tolerance, such as *ompW* and *uspC*, were also up-regulated at human body temperature. Outer membrane protein W is an important outer membrane protein involved in the response to osmotic stress or salt regulation in various bacteria, including *C. sakazakii* [54]. In *E. coli*, under K^+^ starvation or salt stress, UspC can form a complex with KdpD/KdpE to regulate the switch of K^+^ channels and alleviating the stress faced by bacteria [55].

The regulation of gene expression can potentially occur at several stages during the transfer of information from a gene to its coding protein. As for many bacterial processes, post-transcriptional regulation provides a powerful way for the bacteria to rapidly adjust to the changing environment. The post-transcriptional regulatory mechanisms include mainly the activity of RNA-binding proteins, *cis-* and *trans*-acting small noncoding RNAs, and mRNA structures formed in the 5′ UTR [56,57]. Plenty of bacterial sRNAs have been reported to regulate bacterial processes, such as substance metabolism, stress response, virulence factor expression, and biofilm formation, by repressing or activating target gene expression via complementary pairing with target mRNAs [58]. However, identification of sRNA and their regulatory role in *C. sakazakii* remains elusive. Here, 177 sRNA candidates were identified through transcriptome analysis and four of them are highly homologous to previously well studied sRNAs (Table S5). In comparison with the 28 °C group, 64 sRNAs were identified as differently expressed in the 37 °C group, of which 24 were up-regulated and 40 were down-regulated. The difference in expression of these sRNAs indicate their potential roles in adaptation to human temperature and pathogenesis.

We also mapped the transcriptional start site (TSS) and 5′ UTRs. Two diverse classes of 5′ UTR of mRNAs have been exclusively studied: RNA thermometers and RNA riboswitches. These two types of regulatory molecules are complex RNA structures that sense a particular chemical (for riboswitches) or physical signal (for thermometers) and accordingly alter their conformation to control the expression of downstream genes. For example, in *L. monocytogenes*, two vitamin B12-binding riboswitches were identified that control the catabolism of propanediol and ethanolamine molecules through regulating the transcription of non-coding RNAs [59,60]. In *P. aeruginosa*, RNA thermometers were identified to promote the expression level of quorum-sensing transcriptional regulator RhlR at 37 °C [61]. Additionally, the host body temperature (37 °C) has been proved to be a dominant cue to induce the synthesis, assembly, and functionality of the *Yersinia* type 3 secretion system through RNA thermometers at multiple levels [62]. Our results showed that the length of 163 5′ UTRs are longer than 200 nt, which suggests that a significant fraction of the 5′ UTR may function as regulatory RNA elements. Therefore, many post-transcriptional regulatory sequences in the *C. sakazakii* are yet to be discovered.

## 5. Conclusions

According to the results of our study, we can conclude that human body temperature facilitates the growth, acid resistance, osmotic stress resistance, and autoaggregation of *C. sakazakii*, compared to 28 °C. Additionally, RNA-seq analysis between 37 °C and 28 °C revealed 1266 differentially expressed genes, including genes involved in arginine and proline metabolism, carbon fixation, nitrogen metabolism, and several stress tolerance genes (such as universal stress proteins and betaine transporter). We also defined the transcriptional units and analyzed the 5′ UTRs and small RNAs in *C. sakazakii*. Our results offer important insights into the impact of human body temperature on stress tolerance and transcriptome of *C. sakazakii*.

## Figures and Tables

**Table 1:** Strains and plasmids used in this study.

Strain or Plasmids	Genotype or Characteristics	Sources
*Cronobacter sakazakii*		
WT	Wild-type strain *C. sakazakii* ATCC29544	ATCC
Δ*argR*	*argR* deletion mutant	this study
Δ*argACF*	*argACF* deletion mutant	this study
Pasmids		
pKD46	λ red recombinase, Amp^R^	[19]
pKD3	template plasmid for Cm^R^	[19]

**Table 2:** Primers used in this study.

Primer	Primer Sequences (5′-3′)	Amplification Size (bp)
pKD3-*argR*-H1U	CGCAGGAGTGACGGTCCATGAAGGATTACGGTGATTATTCCGTGTAGGCTGGAGCTGCT	1115
pKD3-*argR*-H1D	GAAATGCACACTTTAGCCGCAGGAATCGATTGCTGTGAATAATGGGAATTAGCCATGGT	
pKD3-*argR*-H2U	CGGGCCGGGAAAACAATATCGTTTTTCTTCAACTTTCATCAACGCAGGAGTGACGGTCC	1197
pKD3-*argR*-H2D	TGTCCGGCATTATACGCATGTGCGGTTAGCTGACAAGCAGGAAATGCACACTTTAGCCG	
pKD3-*arcACF*-H1U	ATGCCGCCGTTTTTATTCTGAAAATCAGCCAAGGAATAAATGTGTAGGCTGGAGCTGCT	1115
pKD3-*arcACF*-H1D	TCTCCGAAGCGGCGGGCGCACCGGCGGCGCGCCCGCAAAGGATGGGAATTAGCCATGGT	
pKD3-*arcACF*-H2U	ATCTGCACGGTAACTCGTTATGCGAACACGCGTCGCATAATGCCGCCGTTTTTATTCTG	1196
pKD3-*arcACF*-H2D	ATGGTGTAAGCGGAAGGAAATTTGAACCTGTGCATGAGGGTATCTCCGAAGCGGCGGGC	
pKD3-F	GTGTAGGCTGGAGCTGCT	1033
pKD3-R	ATGGGAATTAGCCATGGT	
*arcC*-F	CGATATTCAGCGTCATAAC	161
*arcC*-R	GAATGTCGAGCGGATAAG	
*argA*-F	GATATTGACACCTTCTCC	160
*argA*-R	TGATGAGTCGTATCTGAT	
*uspC*-F	GGAACTCTTTAATCAAATGT	151
*uspC*-R	TATATTCTCCAGGCTCTC	
*RS21130*-F	GACAAGCAGTACAAGATTA	124
*RS21130*-R	CTTCCTTCTTCTCCTGTT	
*RS21160*-F	TATCAAACAGCCACAATAC	158
*RS21160*-R	CCTTGTCATCTACCTGTG	
*trx-GI*-F	AAGAATGAGCTTCACCTC	81
*trx-GI*-R	GTTGGGATGTTCCGATAG	
*fhuA*-F	GTGAACTTCCTCTATGAC	165
*fhuA*-R	ATCGTTAATCTCAGCAAG	
*proX*-F	CTTTCAAACGGCAATAAC	122
*proX*-R	GTAATCGGCAACTTCATT	
*fecI*-F	TTGATGCAGATGACATTG	160
*fecI*-R	ATCTCCAGATACGCTTTT	
*sdiA*-F	CTGGAAATGAAACTGAGTAAAC	179
*sdiA*-R	GCAGCATAACAGGCAATT	
*csrA*-F	TGACCGTGACAGTTCTTG	104
*csrA*-R	CTGGATACGCTGGTAGAT	
*elaB*-F	ATGCCTTTATCTTCACAA	128
*elaB*-R	GCTTTCAGTTCAACATAC	
*gcvH*-F	TGAAATACAGCAAAGAACA	113
*gcvH*-R	CAGATCGACAAACACCAT	
*narI*-F	GCTGATATTCTGATCCTG	108
*narI*-R	CACCAGTTTCATCATCTC	
*narJ*-F	GAAGAGCAGGTGAAATTC	112
*narJ*-R	CGGCAGAGATATTCAGATA	
*narK*-F	CGGTCAATCTGAATAAGG	172
*narK*-R	CACGGAATAATCAGGATG	
*nirB*-F	GATATGACACGATGACAA	121
*nirB*-R	CCAAAGACGATGATATGA	
*nirD*-F	TATGCCATCAGCAATATC	149
*nirD*-R	CTTTCGTCTTCCATACAC	
*pspG*-F	GTGGCTTTCTTTCTGATG	126
*pspG*-R	CGGCAACACTTTAATCAT	
*uspG*-F	TACAACACCATTCTGATG	188
*uspG*-R	GCCTCTAAATGTTCTTCA	
*dmsB*-F	CTCTCAATAGCCTGTAAC	196
*dmsB*-R	CGTCGCATTTAGTCATAT	
*osmV*-F	GGTAAAGCGGAAGATAAC	131
*osmV*-R	AGATAATCGGCGATATAATC	
*osmW*-F	GATGAATATCGGCGTGAT	152
*osmW*-R	GTAACCAGTCCAGAATAATG	
*osmX*-F	GCTGATTATCTTCAACCA	129
*osmX*-R	AATGCGTAGGTATTGTTC	
*uspA*-F	TGGTAGATAAAGCGGTAT	196
*uspA*-R	GTTTCGGTAATCGGATAG	
*betA*-F	ATCACCTGGAGATGTATC	200
*betA*-R	GAAGTGGTACTGAATGTT	
*betB*-F	GCTTGCAGAAATTTACAC	124
*betB*-R	GGTGAACGAGACTTTATC	
*betI*-F	GCAGGCAGTTAATTGATG	120
*betI*-R	TAAAGTAGTGGCTGATAATG	
*osmY*-F	CTGTTGTATTGGGTTCTG	200
*osmY*-R	GGTGCTCTTAATCTGTTC	

## Data Availability

The original contributions presented in this study are included in the article/Supplementary Material. Further inquiries can be directed to the corresponding authors.

## Supplementary Materials

The following supporting information can be downloaded at: https://www.mdpi.com/article/10.3390/pathogens14030281/s1, Figure S1: Growth curves of C. sakazakii WT strain at 28 °C and 37 °C on TSB and M9; Figure S2: The frequency of operon length; Figure S3: The distribution of UTR length; Figure S4: Identification of Δ*argR*, Δ*arcACF* using PCR; Figure S5: Growth curve of *C. sakazakii* WT strain on LB medium at pH 5.5; Table S1: Growth curve details table; Table S2: Statistical table of gene expression differences; Table S3: Statistical table of up-regulation and down-regulation of gene expression; Table S4: Prediction of transcription start and end sites; Table S5: sRNA prediction.
