# Supplementary material for: Impact of Human Body Temperature on Stress Tolerance and Transcriptome of Cronobacter sakazakii
Source: Pathogens. 2025 Mar 14;14(3):281. doi: 10.3390/pathogens14030281 (PMC11946066; doi:10.3390/pathogens14030281)
Supplement: Supplementary file 1 [file pathogens-14-00281-s001.zip › Supplemental Figures.pdf]

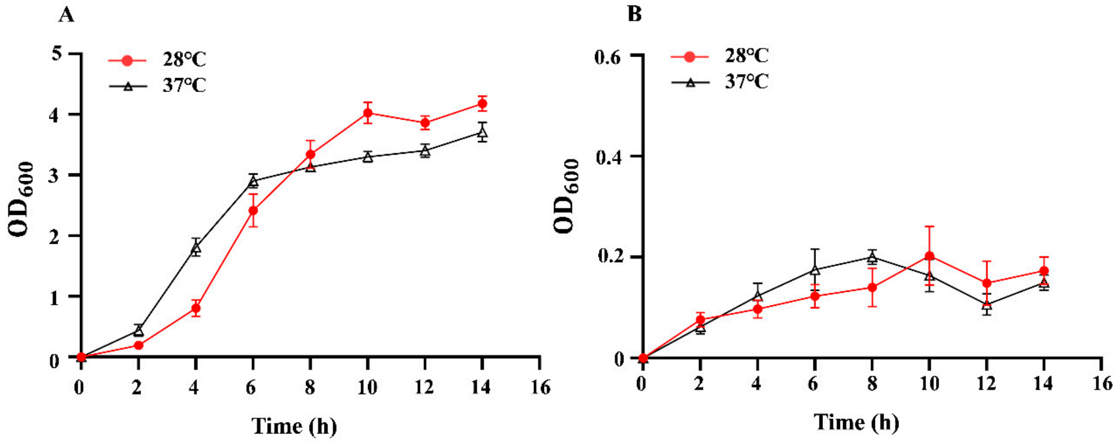

Supplementary Figure 1 Growth curves of *C. sakazakii* WT strain at 28°C and 37°C on TSB and M9. (A)Overnight culture was diluted 100-fold into fresh TSB medium with aeration. The bacteria's optical density (OD) was detected at 600 nm every two hour; (B)Overnight culture was diluted 100-fold into fresh M9 medium with glucose. The bacteria's optical density (OD) was detected at 600 nm every two hour. Error bars represent standard deviations, n=3.

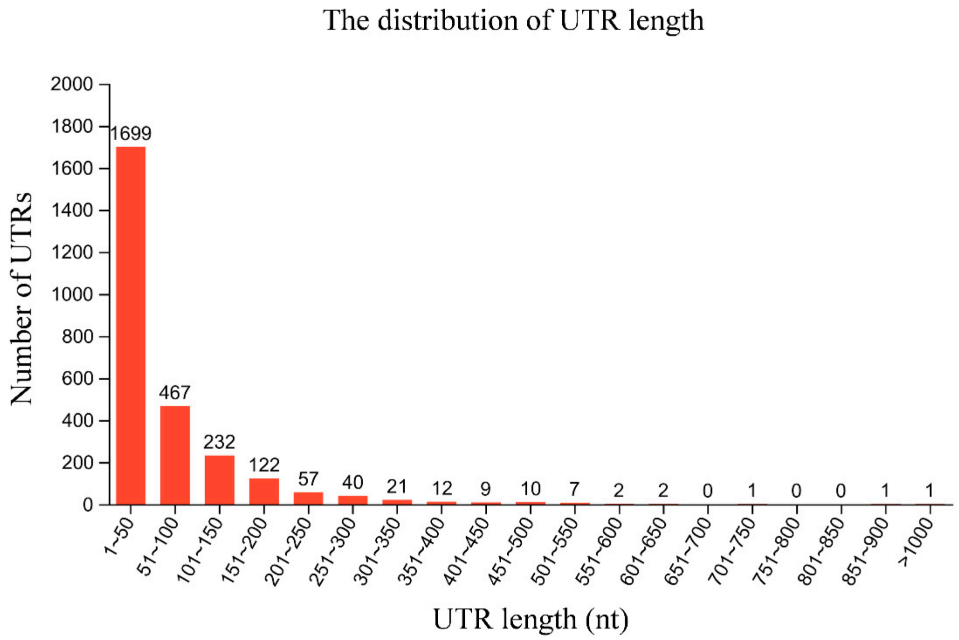

Sup

Supplementary Figure 2 The frequence of operon length. The operon length shorter than 4000bp is over 77%, and only 2.5% are longer than 8000bp.

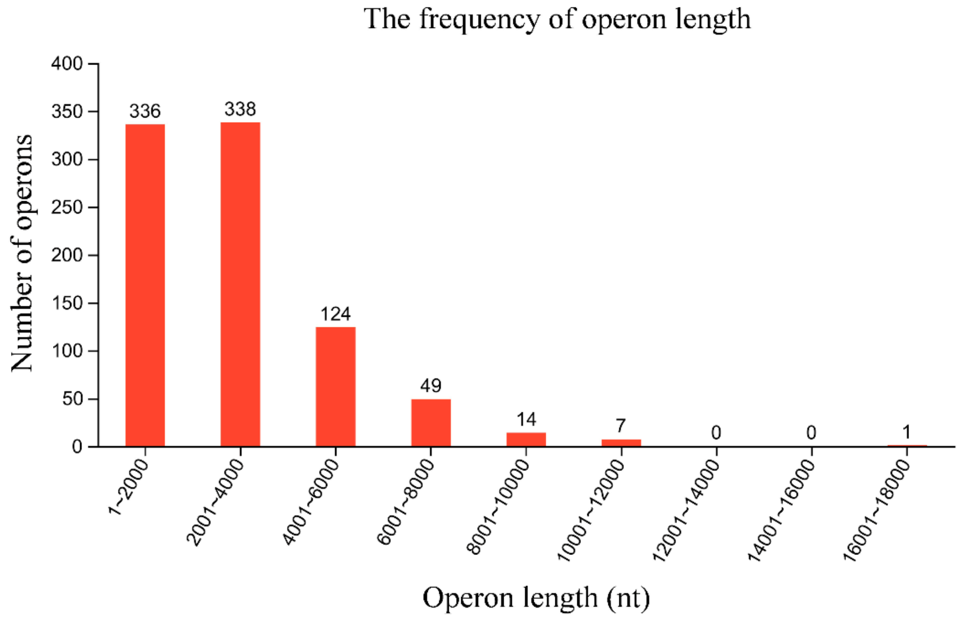

Supplementary Figure 3 The distribution of UTR length. Most of the 5' UTRs (63.3%) were no more than 50 nt and 163 5' UTRs were longer than 200 nt

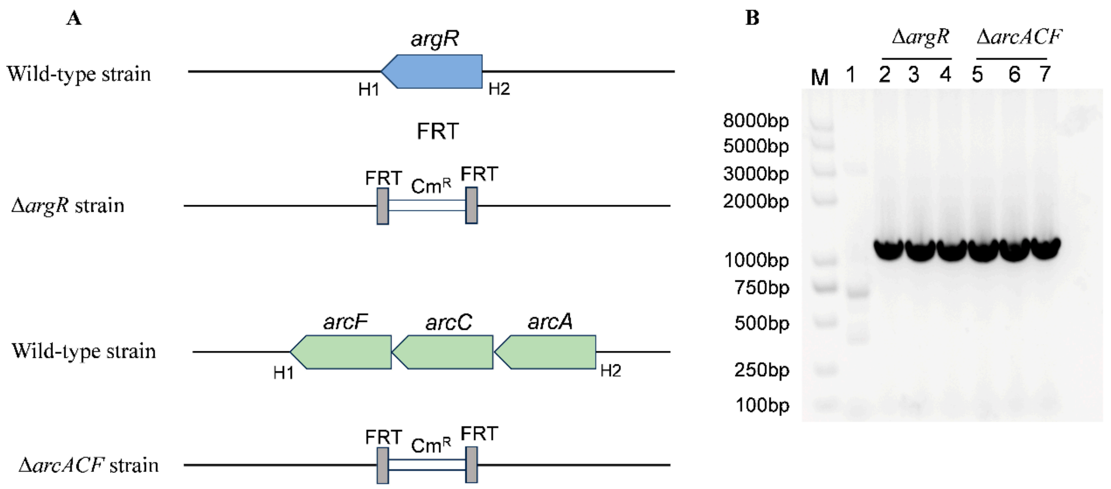

Supplementary Figure 4 Identification of Δ*argR*、Δ*arcACF* using PCR. The gene deletion mutants were constructed using the red recombinase system. (A)The diagram of *argR*, *arcACF* gene before and after deletion. (B) PCR amplification results with the validation primer for Δ*argR* and Δ*arcACF* strains. M, DNA ladder; Lane 1, WT strain; Lane 2-4, Δ*argR* strain; Lane 5-7, Δ*arcACF* strains.

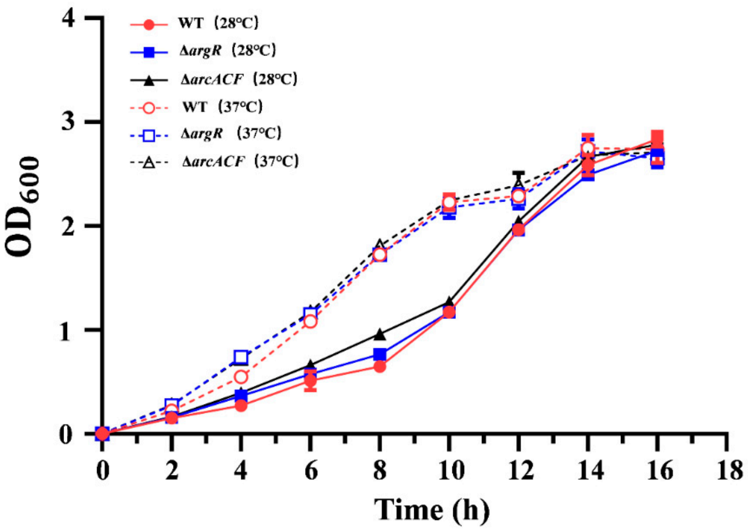

Supplementary Figure 5 Growth curve of *C. sakazakii* WT strain on LB medium at pH 5.5.

The WT strains cultured overnight were diluted at a ratio of 1:100, and then cultured at 37℃ or 28℃on LB at pH 5.5, respectively. OD600 was measured every 2h, which were repeated three times for each sample. Error bars represent standard deviations, n=3.
